# Cultural evolution with uncertain provision of learning resources

**DOI:** 10.1017/ehs.2023.24

**Published:** 2023-10-17

**Authors:** Konstantinos Ladas, Stylianos Kavadias, Jeremy Hutchison-Krupat

**Affiliations:** University of Cambridge, Judge Business School, UK

**Keywords:** Social learning, resource uncertainty, resource allocation, evolutionary stable strategies, cultural evolution

## Abstract

An essential feature of human progress is the use of different modes of learning so agents acquire the appropriate behaviour to survive in a changing environment. Learning may result from agents who discover new knowledge on their own (individual learning), or imitate the behaviour of others (social learning). Social learning is less costly than discovery, but imitation might yield no benefit. Early theoretical models of a population consisting of purely individual and purely social learners found that both types are present in an evolutionary equilibrium. However, the presence of social learners did not provide any improvement to the average population fitness. Subsequent research showed that the presence of social learners could improve the average population fitness, provided that the pure characterisation of the agents’ learning is relaxed. We return to the pure conceptualisation of agents to challenge an assumption in the early work: agents were guaranteed enough resources to perform their desired learning. We show that, if the resources an agent receives are uncertain, this turns social learning into a source of fitness improvement at the population level. Perhaps counter-intuitively, uncertain provision of resources prompts an increase in the proportion of the population that pursues the costlier individual learning activity in equilibrium.

## Graphical abstract

**Figure:**
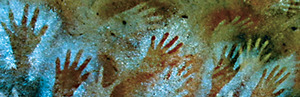

**Social media summary**: Social learning and the uncertain provision of resources. Sufficient aggregate learning resources is not enough; their distribution matters.

## 1. Introduction

The ability to transmit knowledge, behaviours and beliefs through culture is one of the fundamental benefits of being human (Cavalli-Sforza & Feldman, 1981; Boyd & Richerson, 1985; Mesoudi, 2017). Indeed, culture and its evolution are key elements to understand the eventual progress of human societies. The notion of culture itself has many meanings to different audiences (Keesing, 1974; Schein, 2010). Still, despite the various definitions, a common theme is that culture is associated with the ability of individuals to learn from one another. In that respect *social learning* is an enabler of culture (Boyd & Richerson, 1985: 34). Our unparalleled ability to exploit the benefits of social learning has enabled us to adapt far beyond the ability of any other species (Boyd & Richerson, 2005; Henrich, 2001; Mesoudi & Whiten, 2008). For example, few people have spent the necessary learning resources (e.g. time and energy) to prove the Pythagorean theorem. Still, most other people know that given the length of any two sides of a right-angled triangle they can calculate the length of the third. Critically, individuals who have learned this theorem through social learning obtain an answer that is equally correct to someone who has proved the theorem. As such, social learning is of special interest because it is a process that supports cultural inheritance (Wakano et al., 2004). The fact that social learning can yield such a benefit while consuming fewer learning resources has earned it a prominent role in the study of culture.

However, Rogers (1988) put forth a simple yet influential model that challenged the very foundation that social learning plays a role in improving a society's fitness. He considered a population that lived in a temporally varying environment, where a change in the environment implied that a behaviour developed in a prior environment was no longer applicable in the new (changed) environment. Thus, changes in the environment prompted the population to adapt. To study the contribution of social learning, Rogers established two canonical types of individuals in the population. One type, the individual learners, were endowed with an ability to discover the appropriate behaviour for their current environment. However, to learn this new behaviour through discovery required a large quantity of resources (cost). The other type, the social learners, were endowed with an ability to learn new behaviours through ‘imitation’. The only way a social learner could obtain the appropriate behaviour was if they imitated the behaviour of a randomly chosen agent from the previous generation, where ‘imitation’ broadly captures all means of information transmission (e.g. teaching, copying). Imitation required fewer resources for learning than discovery; however, it only worked if the agent who was being imitated had already acquired the appropriate behaviour for the current environment. In other words, while imitation consumed fewer resources, it also came with the risk that the agent would not acquire the desired behaviour.

Rogers’ findings confirmed the belief that social learning was a steadfast component of evolution. Social learners emerged as a stable portion of the population in the long run equilibrium in evolutionarily stable strategies. Yet surprisingly, the presence of social learners did not translate to an improvement in the fitness of the overall population as compared with a population solely consisting of individual learners. This finding challenged the very notion of social learning as a mechanism to improve fitness at the group level, a result sometimes labelled ‘Rogers’ paradox.’ It seemed implausible that given the ‘built-in’ resource efficiency of social learning and the emergence of social learners as a stable portion of the population in equilibrium, that social learning would not have provided a fitness benefit to the overall population. To be clear, the value of social learning was confirmed at the individual level, for any agent endowed with this ability. Rather, the paradox in Rogers’ result revolves around the fact that social learning's value is not reflected at the population level.

Naturally, the above result triggered further study of social learning to investigate whether the apparent paradox was simply an artefact of the model's construction, and to better understand why the benefits at the individual level did not emerge at the population level. As a credit to the original model, changes to the properties of information transmission, and/or the environmental stability, largely served to establish the robustness of the findings. This is not to say the paradox has not yet been addressed, as numerous studies have resolved it (Boyd & Richerson, 1995; Aoki & Feldman, 2014; Enquist et al., 2007; Kharratzadeh et al., 2017). However, the existing resolutions to Rogers’ paradox predominantly focus on the learning approaches employed by the agents. They recognise the ‘pure’ and ‘unsophisticated’ learning mechanisms employed by the agents in Rogers’ model as a common and plausible root cause of the paradox. So long as agents are endowed with some additional sophistication in terms of their decision-making ability, then the resource efficiency of social learning assumed at the individual level also translates to a benefit at the population level.

Thus, while population benefits of the resource efficiency of social learning have emerged, much less attention has been given to the availability of the resources required for learning. We should note, here, that resources for learning are different from the resources required for survival. Resources for survival determine the population size (the carrying capacity if the environment) and they confer a baseline fitness (Cavalli-Sforza & Feldman, 1981; Hofbauer & Sigmund, 1998; Vincent & Brown, 2005). In that regard, they are considered ample once the population size becomes fixed. Resources for learning confer an additional fitness benefit – should the agents acquire the correct behaviour for the current state of the environment. In that light, they have been assumed guaranteed for each agent to perform their learning function, in the literature thus far.

Yet the reality of the situation is that the learning resources available to *each* specific individual may vary. Said differently, even if sufficient learning resources are available to support the population, this does not mean that each and every individual agent is guaranteed to obtain the amount of resources they require to carry out their desired learning function. Some agents could find themselves with excess learning resources, even when other agents are left to suffer the consequences of resource scarcity (Atkinson, 2015). One might even postulate that as societies have progressed from their ancestral foraging past to their complex and hierarchical present, resource disparities have risen (Mattison et al., 2016). Such disparities in learning resources have recently become an even more central topic to societal progress as access to information through formal education and less formal means (e.g. internet) presents a substantial challenge to the priorities of educational institutions, governments and policy-makers (Chaqfeh et al., 2023; Jackson & Holzman, 2020).

Modern societies (from the era of the Mesopotamian civilisation to today's industrialised societies) have required public and private social organisations to balance multiple objectives. Governments must determine how to allocate their resources to education, infrastructure, social programmes, healthcare, the military, etc. Likewise, a private organisation must determine how to allocate their finite budget of resources to research and development (R&D) as opposed to manufacturing or other activities (Si et al., 2022). Critically, for both government and private entities, not all requests for resources will be fulfilled. Moreover, not all activities (R&D vs. manufacturing) face the same odds that their requests will be fulfilled. Naturally, the population-level mechanism for resource allocation (from total resources to resources for specific activities) determines the relative likelihood an individual's request is fulfilled in one area of an organisation as opposed to another. This realisation opens up an interesting research question: could the sheer possibility that an agent might not have sufficient resources to carry out their desired learning enable social learning to emerge and produce a fitness benefit for the population?

Motivated by this question, we return to an original (albeit implicit) assumption in previous work to investigate how uncertainty over the amount of the learning resources available at the agent level affects the overall value of social learning at the population level. More importantly, our analysis and results offer a glimpse into some societal implications of disparate access to learning resources at the individual level.

To model this setting we adopt the original characterisation of agents and the same environmental variability found in Rogers. The only notable change is the uncertainty associated with the learning resources at the individual agent level. We recognise the existence of a multitude of population-level allocation mechanisms that could determine the likelihood an agent eventually has that their learning resource needs will be fulfilled. Our model can accommodate population-level mechanisms where individual learners are prioritised (as captured by a higher relative probability of receiving their required resources) as well as those where social learners are prioritised.

Our focus is the next level. Specifically, a population-level allocation mechanism eventually translates to a random *individual* endowment of resources for each type of agent. From this we derive the equilibrium frequency of social learners in evolutionarily stable strategies, and the associated population fitness. Critically, when learning resources are not guaranteed at the agent level, only a fraction of the population will have sufficient resources to carry out their desired activity in any one generation. In this manner, variability in the availability of resources at an agent level has the potential to affect both individual and social learners and alter the evolutionary dynamics. Our analysis seeks to shed light on how this variability in resource availability relates to the emergence of social learning as both a stable *and* a beneficial component of society.

Our observation that access to learning resources at the agent level is often uncertain reveals an important link to the value of social learning. Provided that each and every agent faces *even the slightest* level of uncertainty over the quantity of resources they could attain, this is enough for social learning to emerge as both a stable portion of the population *and* a mechanism that improves the population as a whole. This result remains true, independent of whether the specific allocation mechanism prioritises one of (or seeks to provide equity between) the different types of agents. Furthermore, this result still holds, even when society is represented by a population of the canonical agents conceived in Rogers’ original model. This is important as it reveals the fundamental value of social learning. The resulting benefit to the population does not require sophisticated agents for it to emerge, and it is evident when the learning resources at the population level are – on average – sufficient to support each agent's learning needs. What matters is that these *resources are not guaranteed at the agent level*.

Additionally, and perhaps surprisingly, we also find that when resources are not guaranteed at the agent level, the population evolves to contain a larger proportion of individuals who pursue the activity that requires *more* resources as compared with a setting where resources are guaranteed. More succinctly, when resources are less likely to be obtained (more uncertain), the equilibrium population contains a greater proportion of individual learners as opposed to social learners. Thus, relatively uncertain resource availability promotes individual over social learning. If we return to the interpretation of individual learners as discoverers, then perhaps our results build on the old adage that ‘necessity is the mother of invention’, as the likelihood of being without learning resources may give rise to a higher relative frequency of independent learners (discoverers) in a society.

## 2. Social learning and its benefit to the population: Insights from prior literature

A Darwinian theory of cultural evolution was formulated in the 1970s and 1980s by Cavalli-Sforza and Feldman (1973, 1981), and by Boyd and Richerson (1985). This theory comprised quantitative models describing the mechanisms by which cultural variations are transmitted within populations. Mesoudi (2017) offers a review of the current state of the theory.

In that context, Rogers (1988) established the possibility that social learning could evolve as a stable component of society but offer no fitness benefit to the group. Numerous studies have scrutinised the parsimony of his model. Boyd and Richerson (1983, 1995) investigate different mechanisms for environmental variability, the ability of agents to know each other's type (biased as opposed to random selection), and the agents’ ability to mix social and individual learning and retain information. They found that Rogers’ result was robust to alternative environmental variation and biased selection; however, once agents exhibited greater learning, sophistication benefits of social learning emerged at the population level. Feldman et al. (1996) incorporated the problem within a replicator dynamics framework to further challenge Rogers’ assumptions that genetic evolution happens far slower than cultural evolution and therefore it can be neglected as a force of change. They too established the robustness of Rogers’ result with regard to the existence of a polymorphic equilibrium that still did not provide any benefit to the group fitness.

Recently, Aoki and Feldman (2014) compiled a comprehensive review of the findings from 11 distinct theoretical models that addressed Rogers’ paradox. They surveyed the evolution of learning strategies in environments that exhibited either spatial or temporal variation. Notably, all of these models established two insights. Firstly, social learning could evolve as a stable component of the population; however, the more resource intensive it is, the less prevalent it becomes. Secondly, they confirmed that despite the resource efficiency of social learning, it still might not improve the average fitness of the population unless the agents have some ‘sophistication’ in their ability to learn (e.g. see discussions in Rendell et al., 2010; Turner et al., 2022). Ultimately, the research on learning mechanisms and the benefits of social learning remains a promising path of inquiry. Still, it remains important to also recognise what has seemingly received scant attention: the availability of learning resources at the individual level, and the likelihood that an individual agent will face a situation of scarcity owing to their uncertain access to resources. Whether social learning affects the mean fitness of the group under such conditions is our path of inquiry.

## 3. A model of social learning when learning resources are uncertain

We seek to understand if (and how) the benefit from social learning at the population level might change once we relax the assumption that agents are guaranteed to obtain the resources they require to carry out their desired mode of learning. We consider agents who require different types of resources: resources to survive and achieve a baseline fitness, and resources to learn – in some way – and possibly obtain a fitness benefit over and above the baseline fitness. Resources for survival determine the size of the population (the carrying capacity of the environment), and in that regard, they are considered ample for a fixed population size. In this work we focus on the implications that might arise when learning resources are not guaranteed at the agent level. In this section we evaluate a setting in which agents face uncertainty over the quantity of resources that they can obtain for their learning function, and we derive how this uncertainty affects the evolution of social learning and its potential to offer a fitness benefit to the overall population.

### 3.1. Key assumptions

A population of agents live in a temporally varying environment which can change between generations with probability, *γ* (0 ≤ *γ* ≤ 1). The parameter *γ* is a measure of environmental volatility; for example, *γ* = 10% implies that the environment changes, on average, once every 10 generations. We assume the environment can take an infinite number of states. If the environment changes then the new environment renders all prior knowledge obsolete. Put another way, a change implies an environmental state that has never been experienced before (Feldman et al., 1996). Each environmental state requires its own uniquely tailored behaviour, where any other behaviour is assumed to be equally ineffective.

Each agent has a baseline fitness, which we assume to be zero. As is customary in evolutionary models, the baseline fitness is a scaling parameter. A baseline fitness of zero might not imply that the agent in question does not survive (Maynard Smith, 1982: 11). Resources that enable the achievement of the baseline fitness are determined by the carrying capacity of the environment. If the agent acquires the appropriate behaviour for their current environment they receive a fitness benefit of one unit over and above the baseline fitness. An agent who does not have the appropriate behaviour receives no fitness benefit beyond the baseline fitness. The agents’ goal is to maximise their fitness, which is accomplished by inferring (learning) the uniquely appropriate behaviour for each environment that they live in. Agents are genetically determined (hard-wired) to be either individual learners or social learners. An individual learner is an agent who can and will only acquire knowledge and behaviour through their own independent discovery. In contrast, a social learner is an agent who can only acquire new knowledge and behaviour through imitation. Social learners imitate a random agent of the previous generation. Naturally, a social learner can only acquire the appropriate behaviour when the agent who they imitate has already acquired the appropriate behaviour for the current environmental state in a prior generation, which is only possible when the environmental state has not changed.

Individual learners expend *c* amount of resources (the individual learning ‘cost’), which guarantees that they acquire the appropriate behaviour for the current state of the environment, and this allows them to achieve a fitness of 1 − *c*. A social learner imitates the behaviour of a random member of the previous generation by expending *s* amount of resources (the social learning ‘cost’). Since social learners are not guaranteed to acquire the appropriate behaviour, their fitness is either, 1 − *s* if they happen to imitate an agent from the previous generation who had already acquired the appropriate behaviour and the environment has not changed, or their fitness is −*s* (i.e. the agent achieves zero benefit but expends the social learning cost). We assume that 1 > *c* > *s* ≥ 0 to reflect the fundamental premise that social learning is efficient from a resources standpoint, also known as the *costly information hypothesis* (Henrich & Henrich, 2007).

We explore the notion that even when learning resources are expected to be sufficient at the population level, this does not necessarily translate to an adequate provision of resources at the agent level. Indeed, variability in the provision of learning resources to individuals (manifested as random individual resource endowments) implies that some agents obtain sufficient resources to perform their desired mode of learning, while others may not. The probability that an agent obtains their required resources is determined by their type. Thus, neither type of agent is guaranteed resources but the likelihood an agent obtains sufficient resources depends on their type.

The manifestation of such an assumption appears straightforward. The reason resources at a population level translate to a random resource endowment at an agent level reflects the reality of private and public organisations alike. Organisations must cater to many diverse, and often conflicting, -objectives. For example, a local government must decide how to allocate their resources to a range of activities (segments) such as healthcare, infrastructure, education, social support, etc. This requires some population-level *allocation mechanism*, where the total resource pool is split amongst the different segments. How the overall pool of resources is split between the different objectives (i.e. population level allocation mechanism) is fairly stable, i.e. it is an infrequent decision (see Si et al., 2022, and references therein). In contrast, within each segment individuals frequently seek to attain resources for their specific initiatives. For example, division managers in private companies regularly meet to consider potential project ideas for the organisation to pursue. Naturally, only some of these are fully funded, and others are not funded at all (Rajan & Zingales, 2001; Harris et al., 1982; Bower, 1972). The lack of guaranteed project funding is captured by a random endowment of resources to each agent.

Rudimentary forms of allocating resources across various objectives, such as public works or military campaigns, can be traced back to elementary budgeting activities in the ancient civilisations of Egypt and Mesopotamia (Eyre, 2013; Silver, 1995). Modern concepts of budgeting in governments began to take shape in Victorian Britain. The British Chancellor of the Exchequer William Gladstone played a significant role in developing the parliamentary budgeting process, where detailed annual budgets were presented to the Parliament for scrutiny (Matthew, 1979). This process was diffused amongst businesses in the early 1920s when James McKinsey, an accounting professor at the University of Chicago and founder of the namesake consulting company, promoted the idea of using budgets as a means to plan and control business operations (McKinsey, 1923). Despite the lack of evidence in support or against the existence of such ‘budgeting’ activities in ancestral societies, it is not beyond reason to expect that some similar process to those of more modern times could have taken place within smaller group settings, such as groups, e.g. how many members should pursue hunting vs. staying behind for the group's protection.

Critically, individuals from different divisions or segments may not face the same probability of obtaining their required resources. The population-level allocation mechanism is meant to reflect the objectives and or standards of a particular society and/or organisation. There is no universally ‘correct allocation’ of resources across all organisational settings. To some ‘correct’ may imply that all individuals have an equal likelihood of obtaining their required resources, independent of which segment or division they are in. To others, the correct allocation might imply that all individuals must draw from the same distribution, such that those individuals with greater needs would have a lower chance of fulfilling their demand for resources. Alternatively, the opposite could be true, where the allocation is based on needs or consumption. We do not argue the benefits or appropriateness of one allocation mechanism or another, rather, our conceptualisation accommodates all of these options and assumes that the population-level mechanism is given. What matters to us is that the individual-level endowment is random and determined by the organisation's group level allocation decision.

We formalise the resource allocation mechanism as follows. We denote by *x* the frequency of the social learners and 1 − *x* the frequency of individual learners in the population. We assume that *R* is the average amount of total resources to be shared among all the agents in the population. We assume that *R* > *c* to ensure that the overall population-level quantity of learning resources is on average sufficient to support all individual agents, *even if* the entire population were individual learners each expending the maximum resources *c*. The total resources *R* must be shared between the two types of agents in the population. Let *λ* be the share of the resources allocated to the *x* social learners, and likewise, 1 − *λ*, be the share of the resources allocated to the 1 − *x* individual learners. In this manner, *λ* characterises the population-level allocation of resources, which directly translates to the likelihood that each type of agent will obtain sufficient resources to carry out their desired learning function. Notably, this conceptualisation of the allocation mechanism is general enough to capture settings when individual (social) learners have a higher probability of obtaining sufficient resources as compared with social (individual) learners, or when either type of agent has an equal probability of obtaining their desired resources. We formally define the ranges of *λ* that correspond to the aforementioned settings below.

Based on the group-level allocation mechanism, individual learners receive a random endowment of resources, *u*_*i*_, which is drawn from a uniform distribution with an interval [0, ^2(1−*λ*)*R*^/_(1−*x*)_]. Likewise, each social learner draws a random endowment, *u*_*s*_, from a uniform distribution with an interval [0, ^2*λR*^/_*x*_]. Our modelling of the random endowment reflects that agents of the same type will *on average* obtain the same resources, where the sum of these individual endowments will, on expectation, equal the share of the total resources allocated to the focal type. A similar setting has been studied by Requejo and Camacho (2011, 2012) in the context of the evolution of cooperation.

### 3.2. Analysis

When resources are uncertain, only a fraction of the population of agents will receive sufficient resources for their desired activity. The probability that an individual learner will be endowed with enough resources, 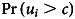, is *δ*_*i*_(*x*) and the probability that a social learner will be endowed with enough resources, 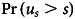, is *δ*_*s*_(*x*), where3.1
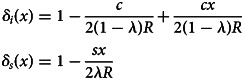
Note that *δ*_*i*_(*x*) is increasing in *x* whereas *δ*_*s*_(*x*) is decreasing in *x*. The intuition for this is as follows: when the resources need to be split between fewer agents, the probability of obtaining sufficient resources increases. In other words, an increase in *x* means fewer individual learners but more social learners, which increases *δ*_*i*_(*x*) and decreases *δ*_*s*_(*x*). We can now formally characterise the allocation point *λ* = Λ_*d*_ where the two types of agents have the same likelihood of obtaining sufficient resources to carry out their desired activity, 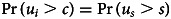, which equivalently translates to *δ*_*i*_(*x*) = *δ*_*s*_(*x*). This yields Λ_*d*_ = *sx*/[*sx* + *c*(1 − *x*)]. Consequently, when *λ* < Λ_*d*_ (*λ* > Λ_*d*_) it implies an individual (a social) learner will have a higher probability of obtaining their required resources than a social (an individual) learner would.

Let *q*(*x*) denote the frequency of tuned agents in the current generation, i.e. agents who have acquired the appropriate behaviour for the current state of the environment. Assuming the frequency of the tuned agents in the next generation, to be *q*^′^(*x*) we can derive the recursion that governs the evolution of the tuned population. If the environment does not change in the next period, an event with probability 1 − *γ*, then the frequency of tuned agents in the next generation, *q*^′^(*x*), will comprise all of the individual learners with enough learning resources, and the fraction of social learners with enough learning resources who copied a tuned agent from the current generation. If the environment changes, an event with probability *γ*, only the individual learners who have sufficient learning resources will be tuned to this new environment and all social learners become non-tuned. Therefore, the frequency of the tuned agents in the next generation, *q*′, is:3.2
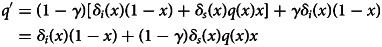
The fixed point q*(*x*) of the above recursion is3.3
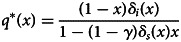


When access to resources is uncertain, not all individual learners are able to acquire the appropriate behaviour. Specifically, the fraction 1 − *δ*_1_(*x*) of them who do not obtain sufficient resources are unable to perform their learning function. We assume that these individual learners get the baseline fitness of zero (an assumption we relax in Section 4). Therefore, the average fitness of individual learners depends on the *fraction* of individual learners who obtain sufficient resources. The average fitness of the individual learners is then3.4
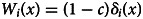


Following a similar logic, a social learner's ability to acquire the appropriate behaviour depends on the number of tuned agents in the population *and* the availability of sufficient resources. Thus, a social learner's expected fitness is (1 − *γ*)q*(*x*) − *s* when they have sufficient resources available (an event with probability *δ*_2_(*x*)), and zero if they are unable to obtain sufficient resources. Hence, the average fitness of the social learners is:3.5
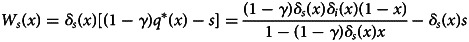


The evolutionary stable strategies (ESS) condition dictates that the long-run equilibrium frequency of social learners, x*, satisfies *W*_*i*_(x*) = *W*_*s*_(x*) (Maynard Smith, 1982; Weibull, 1997; Hofbauer & Sigmund, 1998). Therefore, x* is given by3.6
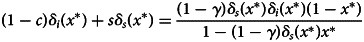


At the equilibrium frequency x* > 0, the values of fitness of the individual and social learners are equal and therefore the mean fitness of the population 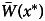 is 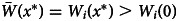. Hence, social learning offers an improvement to the mean fitness at the population level. This holds when the condition *W*_*s*_(0) ≥ *W*_*i*_(0) is satisfied, which translates into an upper bound for the splitting parameter *λ*, given by3.7
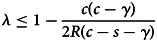


**Figure 1.**
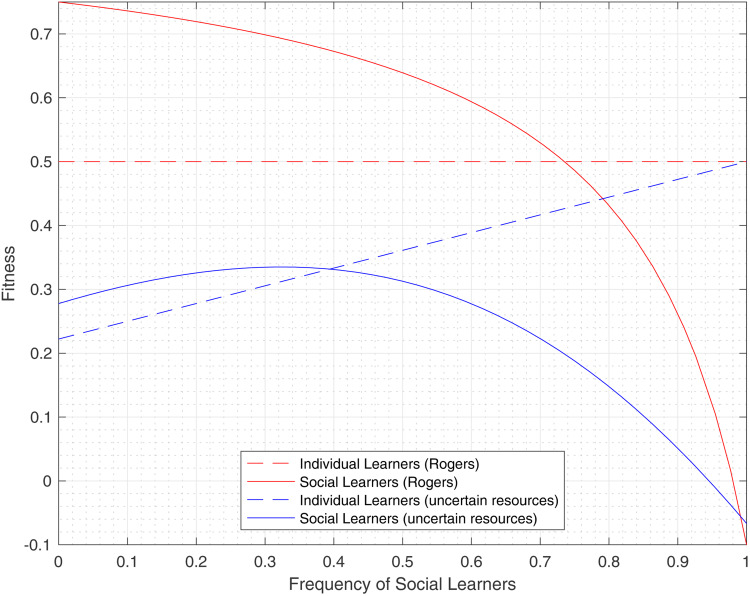
Fitness of individual and social learners as a function of social learners’ frequency. In this example *γ* = 0.10, *c* = 0.50, *s* = 0.20, *R* = 0.60 and *λ* = 0.25. Red lines are the guaranteed resources model (Rogers) and blue lines are the uncertain resources model. The ESS frequency is at the intersection of individual learning and social learning curves.

Figure 1 provides a graphical depiction of the equilibrium fraction of social learners when the access to the resources at the individual agent level is guaranteed (red colour) or uncertain (blue colour); the individual (social) learners’ fitness is represented by dashed (solid) lines.

It is important to note under what conditions our model will revert to the same results as derived in the original Rogers model (Rogers, 1988). The condition is straightforward: when the overall population resources are large enough (*R* → ∞) to guarantee individual agent access to their required resources (*δ*_*i*_(*x*) → 1 and *δ*_*s*_(*x*) → 1). When *R* → ∞ then the fixed point of tuned agents of (3.3) becomes 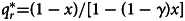, the fitness of social learners of (3.5) becomes 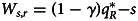, the fitness of individual learners is *W*_*i*,*r*_ = 1 − *c*, and the equilibrium frequency of social learners becomes3.8
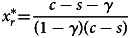


Note that 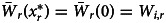, i.e. social learners do not improve the overall population's average fitness. This last observation is a key result of Rogers’ (1988) paper. However, note that our derivation is different because we analyse an infinite environmental states setting.

There is another, less obvious, condition for which our model also reverts to the Rogers result. It emerges from a specific division of the population resources: when the percentage of the overall population resources allocated to social learners, *λ*, is exactly the same as the fraction of social learners x*, then the distributions from which each type of agent draws their resource endowment are the same. In this special case, the probability that each agent obtains their required resources becomes independent of the frequency of each type of agent. When this happens, our model produces the same results as the Rogers analysis, i.e. the presence of social learners do not provide a benefit to the fitness of the overall population.

**Figure 2.**
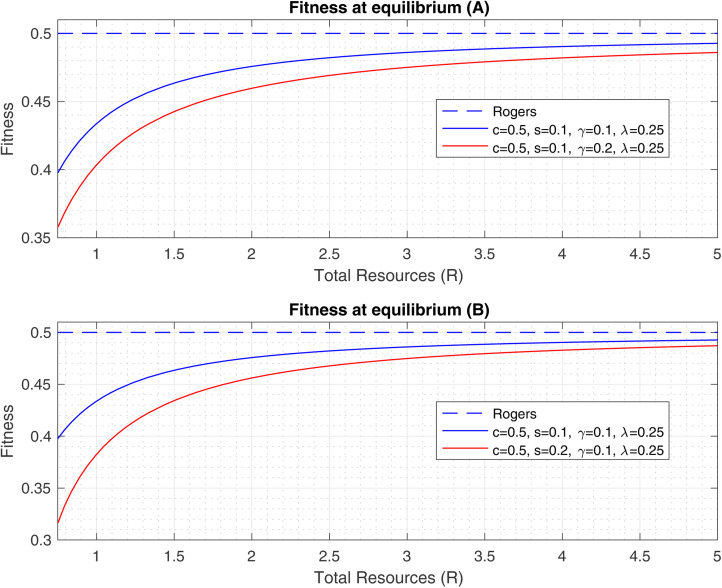
Fitness at equilibrium as a function of the expected population level resources, *R*. The dashed blue line shows Rogers’ equilibrium fitness (invariant to all parameters but *c*), the solid blue lines represent the baseline of *c* = 0.50, *λ* = 0.25, *γ* = 0.1, and -*s*- = 0.1 and the red line depicts (A) a 0.1 increase in *γ* and (B) a 0.1 increase in *s*.

Figure 2 depicts the mean fitness of the population at equilibrium as a function of the maximum total resources allocated to the population *R* for various choices of the environmental variability *γ* and the cost of social learning *s*. We also show how the population fitness converges to the Rogers fitness at equilibrium in the limiting case when *R* is large enough.

### 3.3. Results

In a settings where the provision of resources is uncertain and agents might not obtain the resources required to carry out their desired learning function, two fundamental results emerge. Firstly, the mean population fitness at equilibrium, x*, is larger than the mean fitness at *x* = 0. Specifically, we find that 
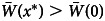
, which resolves Rogers’ paradox (Boyd & Richerson, 1995). This is because the ESS condition requires *W*_*i*_(x*) = *W*_*s*_(x*), which implies that 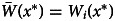 and x* > 0 while *W*_*i*_(*x*) is increasing in *x*. Therefore, when resources for learning are uncertain, social learning improves the mean fitness of the population. What matters, though, is *why*.

When the quantity of resources an individual obtains is uncertain, each additional social learner provides an indirect benefit to all of the individual learners. This benefit comes about simply because any increase to the frequency of social learners implies a decrease in the proportion of individual learners, which means each individual learner has a *higher* probability of obtaining their required resources for their desired learning function. Ultimately, a higher probability to obtain resources translates directly into a higher fraction of the individual learners being able to carry out their desired learning as indicated in equation (3.1) and a higher expected fitness as indicated by equation (3.4).

This interplay between the frequency of social learners and the probability of generating fitness reveals the potential value of social learning at the population level. That is, even when the agents lack the sophistication to choose their desired mode of learning and they lack the ability to retain prior learning, the simple possibility that an agent *might* not obtain sufficient resources is enough to enable social learning to provide a benefit to the group.

Secondly, we find that uncertain resource provision at the individual level prompts the equilibrium frequency of *individual learners* to be *higher* than it would be if resources for learning were guaranteed, 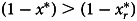. At first, this appears surprising. After all, individual learning requires *more* resources (is the more costly activity). Therefore, given uncertain resources one might suppose that the more costly activity should experience a *decrease* in its frequency. Yet, despite individual learning being more costly, evolution that takes place under uncertain resource provision favours individual learners over the more ‘cost effective’ social learners. Upon reflection, this does make intuitive sense: a critical mass of individual learners *must* exist for social learners in subsequent generations to even have an opportunity to imitate the appropriate behaviour. Moreover, since not every one of the individual learners will have sufficient resources to obtain the appropriate behaviour, the frequency of individual learners required to achieve this critical mass should be even higher. The mathematical details of this result are provided in Appendix (A.2).

Importantly, the splitting parameter *λ* allows us to consider a vast range of population-level allocation mechanisms, from those that prioritise individual learners 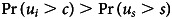 to those that prioritise social learners 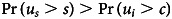 and everything in between. Across the full continuum, there is a single point where the results for an uncertain provision of resources do not differ from the outcome of Rogers. This unique point is when *λ* = x*, that is the overall population resources are divided proportionally to the equilibrium frequencies of learners. For any other *λ*, independent of whether 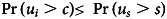, we consistently find that 
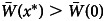
.

## 4. A model of social learning with alternative sources of fitness

In this section we relax the assumption from Section 3.2 that agents who lack the required resources cannot obtain a fitness beyond the baseline (zero) fitness. To clarify, agents who fail to obtain sufficient resources for their desired learning function remain unable to perform this function; however, we now allow them to pursue a different path and a possibility to achieve a level of fitness beyond the baseline. That is, we recognise that an agent's desired learning function is not the only way to obtain a non-zero fitness; agents may have alternative means through which they can generate *some* fitness above zero. The critical assumption is that this alternative means is possible even when an agent has not obtained sufficient learning resources for their intended learning function. This relaxation ensures learners are not severely ‘punished’ for not having obtained their required resources and while maintaining all other elements of the main model as we detail below.

Consistent with the model presented in Section 3.1, individual learners (a fraction 1 − *x* of the population) require *c* resources to obtain a fitness of 1 − *c*. However, if their random endowment is insufficient to support such an activity (an event that occurs with probability 1 − *δ*_*i*_), then their next best option is to pursue an alternative source of fitness. This alternative source provides an opportunity to achieve a *semi-tuned* state, which yields a fitness of *ω*, where naturally this alternative source provides a fitness level that is less than what could have been obtained if they had sufficient resources, *ω* < 1 − *c* < 1 − *s*. Moreover, we recognise that even the fitness level, *ω*, might still not be guaranteed. Agents who pursue this alternative source achieve a semi-tuned state with an exogenous probability *z*, where *z* ∈ [0, 1], otherwise they remain in an out-of-tune state with probability 1 − *z*. In this manner, if *z* = 1 all agents with insufficient learning resources are guaranteed to be semi-tuned, and when *z* = 0 the model reverts back to the model of Section 3.2. As before, out-of-tune agents remain at a baseline fitness of zero. We summarise how the various states of individual learners translate into their respective levels of fitness in Table 1.

**Table 1.** Fitness of the individual learners.

Individual learners	State	Fitness
*δ*_*i*_	Tuned	1 − *c*
(1 − *δ*_*i*_)*z*	Semi-tuned	*ω*
(1 − *δ*_*i*_)(1 − *z*)	Out-of-tune	0

The same logic applies to the social learners. We assume that social learners (a fraction *x* of the population) require *s* resources to copy a random agent from the previous generation. Let *δ*_*s*_ be the fraction of social learners with enough resources. The next best option for a social learner with insufficient resources (an event that occurs with probability 1 − *δ*_*s*_) is to pursue the same alternative source of fitness that is available to individual learners. The fitness a social learner acquires depends on who they copy and whether the environment has changed or not. Table 2 provides the respective fitness levels and possible states for social learners when the environment has not changed. When the environment changes, social learners become out-of-tune independently of who they copy.

**Table 2.** Fitness of the social learners when the environment does not change. The column ‘Copy’ denotes the state of the agent from the previous generation whom the focal social learner imitates, when he/she has the resources to perform their learning function.

Social learners	Copy	State	Fitness
*δ*_*s*_	Tuned	Tuned	1 − *s*
*δ*_*s*_	Semi-tuned	Semi-tuned	*ω* − *s*
*δ*_*s*_	Out-of-tune	Out-of-tune	−s
(1 − *δ*_*s*_)*z*	–	Semi-tuned	*ω*
(1 − *δ*_*s*_)(1 − *z*)	–	Out-of-tune	0

We denote the frequency of tuned agents by *q* and the frequency of semi-tuned agents by *p*. Following the same procedure as performed in Section 3.2 we compute the fixed points for the tuned agents q* and semi-tuned agents p* as given below (detailed derivations are provided in Appendix A.1):4.1
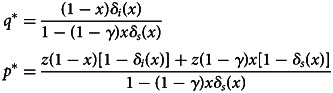
The fitness of the individual learners, *W*_*i*_(*x*), and the social learners, *W*_*s*_(*x*), depend on the fixed points q* and p* as shown below (detailed derivations are provided in Appendix A.1):4.2
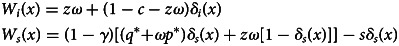
The equilibrium point x* satisfies the equation *W*_*i*_(x*) = *W*_*s*_(x*). Note that *ω* < 1 − *c* and 0 < *z* < 1, hence *zω* < 1 − *c*. Then, because *δ*_*i*_(*x*) is increasing in *x*, it follows that ∂*W*_*i*_/∂*x* > 0. Importantly, even when agents have an alternative source to acquire fitness beyond the baseline level, we still arrive at the same qualitative conclusion as Section 3.2. This provides further evidence on the robustness of the result. Specifically, when the provision of resources is uncertain at the individual level, social learning can provide an improvement to the fitness at the population level, even if agents with insufficient resources are guaranteed to achieve *some* non-zero fitness benefit through some alternative sources of fitness.

**Figure 3.**
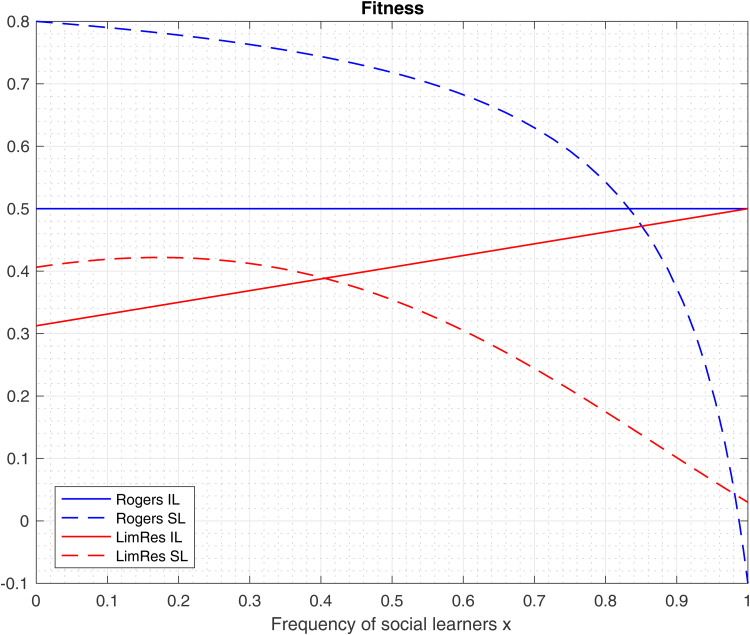
Fitness of individual and social learners as a function of social learners’ frequency. In this example *γ* = 0.10, *c* = 0.50, *s* = 0.10, *R* = 0.60, *λ* = 0.17, *z* = 0.50 and *ω* = 0.25. Blue lines are the guaranteed resources model (Rogers) and red lines are the uncertain resources model with semi-tuned agents. The ESS frequency is at the intersection of individual learning and social learning curves.

In Figure 3 we depict the fitness of individual and social learners when they pursue an alternative source of generating fitness as compared with the respective fitness of the guaranteed resources model (Rogers). As in the model of Section 3.2, the population reaches an equilibrium fitness higher than that when social learners are absent from the population (*x* = 0), indicating the robustness of the result in Section 3.2, 
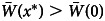
.

Overall, for the vast majority of population level allocation mechanisms, some uncertainty in the provision of resources to individuals is enough to enable social learning to offer a fitness benefit to the population, even when agents do not solely rely on learning as a source of fitness. Moreover, while social learning offers a fitness benefit to the population, it also lends further importance to the role of individual learners. Said differently, uncertain resource provision requires an even greater foundation of individual learners, despite the burden on resources they might impose.

## 5. Discussion

In an influential study, Rogers (1988) showed that social learning could be a steadfast component of a population without providing any improvement to the mean fitness of the population. The fact that social learning would not offer a population-level benefit has been discussed in the literature (see Aoki & Feldman, 2014, and references therein) and resolved, largely by adaptations to the agent's learning abilities. That is, boundary conditions on Rogers’ result have been provided in terms of what the agents can decide and what they can retain. One element that has been largely left untouched, though, is the provision of resources at the agent level.

We show that, even in a population of canonical agents who perform learning as in Rogers’ original model, uncertainty over the quantity of learning resources at the individual level enables social learning to become a potent force for improving the mean fitness of the population. Uncertainty over the provision of resources at the individual level is a common occurrence in public and private organisations, as it has been over many centuries, e.g. resource access in Mesopotamian civilisation (Silver, 1995). Given an overall set of resources, public and private organisations alike employ established allocation mechanisms to split these resources between the different activities as they seek to fulfil multiple objectives. In this manner, these allocation mechanisms partition the organisation's total resources (‘their budget’) to a set of activity level allocations. Naturally, the magnitude of resources allocated to each activity determines the likelihood that individual activities can be pursued. In other words, individual agents may experience uncertain access to resources that appear as random endowments. Moreover, agents may not face the same probability that their individual activity is pursued.

We model these resource allocation mechanisms as a split of the total learning resources at the population level between the two focal types of learning functions. Our split allows us to capture a broad range of allocation mechanisms within a group, which may (non-exhaustively) reflect the objectives of the organisation, the power structure, status, etc. at the group level. For example, a business organisation may give priority to one type of activity or another (e.g. environmental, health and safety as opposed to R&D), independent of whether it is more or less costly (Si et al., 2022). Our conceptualisation allows us to consider settings where a high-cost activity has a higher probability of acquiring the necessary resources, and likewise, settings where low-cost activities may get priority. We model each individual's resource provision as random individual endowments, where the prioritisation of activities is reflected in each type of individual's likelihood of obtaining sufficient resources. This allows us to capture the possibility of resource scarcity at the individual level, even when the population-level (total) resources are on average (more than) sufficient to support the group.

Our perspective is novel and complements earlier studies which inform our understanding of how social learning can improve the mean fitness of a population. Importantly, we conceptually depart from these prior studies in terms of our focus. We do not pursue the rationale that agents must hold more elaborate and sophisticated learning abilities (e.g. selective or cumulative learning) to explain how social learning can create a benefit for the population. Instead, we show how the existence of random individual endowments for different types of agents is enough to enable social learning to deliver a fitness improvement at the population level.

Our model also shows how uncertainty, and the possibility of scarcity, with regard to learning resources at the individual level, shifts the equilibrium ratio of individual to social learners, in a somewhat counter-intuitive way. Specifically, given the existence of a population-level allocation mechanism that splits (prioritises) resources between activities and a random individual endowment, the proportion of individual learners within the population increases. Said differently, evolutionary forces prompt the population to favour the discovery of new knowledge as opposed to relying on the (cheaper) diffusion of existing knowledge when learning resources are uncertain at the individual level. While Rogers’ original model captures that new knowledge must be generated for there to be any benefit of diffusion, our model shows how the necessity of new knowledge is even *more* important when resources are uncertain at the individual level.

We also incorporate the possibility that agents improve their fitness even when they are not endowed with sufficient learning resources. Specifically, we give agents the possibility to acquire a fitness above their baseline fitness, even when they are not endowed with sufficient resources to carry out their desired learning activity. So long as they are not guaranteed to achieve a fitness equivalent to what an individual learner with sufficient resources could achieve, all of our results continue to hold.

In conclusion, our study may open up discussions on two broader important issues: first, the crucial role that resource allocation mechanisms play within populations with respect to the overall population performance – disparities in resource provision may end up having profound effects on social performance; and second, the potential value of evolutionary modelling to analyse managerial and economic contexts. Further studies should try to address a wider breadth of phenomena within management, industrial organisation and economics. This should happen in the same spirit as with models of cultural evolution, which were initially developed in anthropology and biology (Boyd & Richerson, 1985), but have increasingly been used to study the evolution of human behaviour (Axelrod & Hamilton, 1981; Mesoudi, 2017; Turchin, 2018), to examine technological accumulation (Boyd et al., 2013) and to advance our understanding of cultural dynamics in psychology (Pan et al., 2021).

## Supporting information

Ladas et al. supplementary material 1 Ladas et al. supplementary material

Ladas et al. supplementary material 2 Ladas et al. supplementary material

## Data Availability

N/A.

## Supplementary material

The supplementary material for this article can be found at https://doi.org/10.1017/ehs.2023.24.

## Research transparency and reproducibility

No data, materials or software protocols were involved for the execution of the study.

## Appendix A

### A.1. Agents with richer behaviour

Let's denote by *q* and *p* the frequency of the tuned agents and semi-tuned agents in the current generation and by *q*^′^ and *p*^′^ the same frequencies in the next generation. If the environment changes, tuned agents are the only individual learners with enough resources. If the environment stays the same then tuned agents are the individual learners with enough resources along with the social learners that have copied a tuned agent. Therefore, the dynamics of *q* are governed by the equation:

which simplifies to

The fixed point of the recursion is

A.1

The dynamics of *p* are governed by the recursion

A.2

The fixed point of this recursion is then

A.3

The fitness of the individual learners is

A.4

The fitness of the social learners is

A.5

### A.2. ESS frequency under resource constraints is smaller than the the ESS frequency under no constraints

Let *W*_*s*,*r*_(*x*) and *W*_*s*,*c*_(*x*) denote the fitness of social learners under no constraints or limitations (the original Rogers model) and the fitness of social learners under constraint limitations respectively. These fitnesses are given by:

A.6

A.7

with *δ*_*i*_(*x*) < 1 and *δ*_*s*_(*x*) < 1 for all *x*. The exact form of *δ*_*i*_ and *δ*_*s*_ are given in equation (3.1). The fitness of individual learners under the Rogers model and that under the constraint model are *W*_*i*,*r*_ = 1 − *c* and *W*_*i*,*c*_(*x*) = *δ*_*i*_(*x*)(1 − *c*) respectively.

Let

We can write then the fitnesses in terms of the expressions *G*_1_ and *G*_2_ as *W*_*s*,*r*_(*x*) = *G*_1_(*x*) − *s* and *W*_*s*,*c*_(*x*) = *G*_2_(*x*) − *sδ*_*s*_(*x*). Let *x** be the ESS equilibrium of the constraint problem. The equilibrium *x** satisfies *W*_*s*,*c*_(*x**) = *W*_*i*,*c*_(*x**) = *δ*_*i*_(*x**)(1 − *c*), which implies that *G*_2_(*x**) = *δ*_*i*_(*x**)(1 − *c*) + *sδ*_*s*_(*x**). Note that

Because *δ*_*s*_(*x**) < 1 then *k*(*x**) < 1. Rearranging terms in the last two equations we find that

Because *δ*_*i*_(*x**) < 1, *δ*_*s*_(*x**) < 1, and *k*(*x**) < 1 then *W*_*s*,*r*_(*x**) > 1 − *c*. The derivative of *W*_*s*,*r*_ with respect to *x* is

Hence *W*_*s*,*r*_(*x*) is decreasing in *x*. Because Rogers’ equilibrium point  satisfies , it follows that .
